# A Carriage-Structured Model of Meningococcal Meningitis: Backward Bifurcation, Seasonal Recurrence, and the Reach of Conjugate-Vaccine Strategies in the African Meningitis Belt

**DOI:** 10.3390/epidemiologia7050140

**Published:** 2026-10-08

**Authors:** Siphokazi Princess Gatyeni

**Affiliations:** Department of Mathematics and Applied Mathematics, University of Johannesburg, Johannesburg 2006, South Africa; siphokazig@uj.ac.za

**Keywords:** meningococcal meningitis, nasopharyngeal carriage, backward bifurcation, centre manifold, seasonal forcing, conjugate vaccine, meningitis belt

## Abstract

Background: Epidemic meningococcal meningitis in the African meningitis belt recurs on two timescales: a sharp annual dry-season peak and irregular multi-annual epidemics five to twelve years apart, whose mechanistic origin remains unsettled. Invasive disease is a rare, dead-end outcome of asymptomatic nasopharyngeal carriage, so susceptible–infectious templates misrepresent community transmission. We ask how carriage structure shapes bifurcation, seasonal recurrence, and the reach of conjugate-vaccine strategies. Methods: We formulate a deterministic carriage-structured model in which only carriers transmit, immunity against re-acquisition is leaky, and a conjugate vaccine is imperfect and wanes. We use the Castillo-Chavez–Song centre manifold method, numerical continuation, Floquet and eigenvalue analysis, and scenario simulations of abstract rate- and pulse-type interventions parameterised from published belt ranges. Results: The basic reproduction number $R_{0}$ is a property of carriage transmission, decoupled to first order from disease incidence. Backward bifurcation at $R_{0}=1$ is governed by a threshold $\varepsilon^{*}$ on carriage-blocking immunity; only the untreated fatality hazard and treatment capacity are absent from the local condition. It is impossible without vaccination and, with any positive routine coverage, persists across the plausible coverage range, giving vaccine-induced bistability. Continuation confirms a fold at $R_{0,c}\approx 0.946$ ($\varphi =0.12\;{\mathrm{yr}}^{-1}$, weak carriage immunity), but not at baseline immunity. Under seasonal forcing, dynamics converge to a simple annual cycle throughout the region searched; we found no multi-annual recurrence, although damped eigenmodes of the unforced system have periods overlapping the observed interval. Conclusions: Within this deterministic, single-strain model, irregular recurrence likely requires additional structure, with stochastic amplification of damped cycles a natural next step. Whether sustained immunisation eliminates epidemics or merely postpones them may depend on the same carriage-efficacy threshold that governs the bistability result.

## 1. Introduction

Bacterial meningitis remains a leading cause of death and long-term neurological disability in sub-Saharan Africa. The African meningitis belt, a band of twenty-six countries stretching from Senegal to Ethiopia, experiences a characteristic epidemiology found nowhere else: hyperendemic transmission of *Neisseria meningitidis* punctuated by explosive dry-season epidemics and, over longer horizons, by irregular multi-annual waves recurring every five to twelve years [1,2,3]. The introduction of the monovalent serogroup A conjugate vaccine MenAfriVac from 2010 virtually eliminated serogroup A disease [4], but epidemics caused by serogroups C, W, X and Y have continued, and a pentavalent conjugate vaccine (Men5CV/MenFive) has since been prequalified and recommended for introduction across the belt [5].

Two features of meningococcal biology make the disease unusual to model. First, transmission is carried almost entirely by asymptomatic nasopharyngeal carriage; invasive disease is a rare complication of carriage on the order of one per cent of carriage episodes or fewer, and invasive cases do not themselves add materially to the population-level force of infection [6,7]. Meningococci recovered from blood and cerebrospinal fluid in confirmed cases are genetically indistinguishable from strains found in nasopharyngeal carriage, consistent with invasion being a within-host progression of an existing carriage episode rather than a distinct transmissible state [6]; secondary cases clustering around an index case are attributable to shared exposure to the same carried strain rather than to transmission from the diseased individual [6,7]. A model in which the notified case is treated as the transmitting unit therefore describes the wrong process for the airborne, community-level spread that drives belt epidemics, even though contact tracing and post-exposure prophylaxis around an index case remain sound practice at the household scale, where shared exposure to the same strain elevates carriage risk in a way a homogeneously mixing model does not represent.

Second, the recurrence of epidemics on the multi-annual timescale is not mechanistically settled: candidate explanations include seasonal forcing of the transmission rate through Harmattan dust and low humidity, seasonal forcing of the invasion rate through mucosal damage or transient co-infection, and the slow accumulation and waning of population immunity [3,8]. Irving et al. [9] showed by simulation that seasonal forcing of transmission alone cannot reproduce the observed irregular multi-annual dynamics, and that waning immunity or an analogous slow process is required. That finding has not since been revisited with the tools of bifurcation theory.

Transmission-dynamic models have been used to evaluate conjugate-vaccine strategies in the belt [10,11], most recently to assess multivalent-vaccine rollout, with vaccine effectiveness against carriageidentified as the critical remaining unknown [12]. Here we take a complementary route: rather than fitting a detailed simulation model, we build a parsimonious carriage-structured model whose structure is transparent enough to analyse, and we ask what its equilibrium and bifurcation structure imply for prevention. Our contributions are: (i) a demonstration that, within this model, $R_{0}$ is a carriage quantity, essentially decoupled from disease incidence (Proposition 2); (ii) a closed-form local backward-bifurcation condition, obtained by the centre manifold method, together with a proof that backward bifurcation is structurally impossible in the absence of vaccination and requires routine coverage to become possible (Theorem 2, Corollary 1, Corollary 2), confirmed numerically by equilibrium continuation; (iii) a Floquet analysis of the seasonally forced model together with an explicit, computed classification of its attractor structure over immunity-waning and seasonal-amplitude space, which finds simple annual dynamics throughout the region searched, and a structural argument for why; and (iv) a scenario analysis linking the carriage-efficacy threshold to whether sustained immunisation may eliminate or merely postpone epidemics.

## 2. Model Formulation

We partition a homogeneously mixing population of constant size *N*, normalised to unity, into five compartments: susceptible *S*; vaccinated *V*; asymptomatic carriers *C*; individuals with invasive disease *I*; and recovered/immune *R*. Only carriers transmit. The force of infection is

(1) λ(t)=β(t)C,

where β(t) is the (possibly seasonally forced) transmission rate. New carriage acquisition draws from the susceptible pool, from the vaccinated pool at reduced rate (1−σ), and from the immune pool at reduced rate (1−ε).

**Remark** **1** (Terminology: σ versus ε, and what “leaky” means)**.** *Two distinct protective mechanisms are represented, and we use them consistently throughout.* Vaccine efficacy against carriage*, σ∈[0,1], is the proportional reduction in the rate at which a* vaccinated *individual acquires carriage relative to an unvaccinated susceptible; a vaccine is* leaky *when σ<1, i.e., it lowers but does not abolish the acquisition hazard, in contrast with an all-or-nothing vaccine that would fully protect a fraction σ of vaccinees and leave the remainder unprotected.* Carriage-blocking immunity after clearance*, ε∈[0,1], is the analogous proportional reduction in acquisition hazard for individuals in the recovered/immune class R. Because protection against meningococcal carriage following natural clearance is serogroup- and strain-specific and does not durably block re-acquisition of a heterologous or even homologous strain [6,7], we likewise treat this immunity as leaky (ε<1) rather than sterilising: longitudinal carriage surveys show that acquisition and clearance of N. meningitidis carriage recur across the life course, including in individuals who have previously cleared a genetically indistinguishable or serogroup-matched strain, and that carriage prevalence and turnover remain substantial in older, previously exposed age groups rather than falling to zero as sterilising immunity would predict [7]. The two parameters are structurally parallel (both multiply the acquisition hazard λ) but act on different compartments (V versus R) and are estimated from different data: vaccine-probe carriage trials for σ, longitudinal carriage acquisition/clearance studies in previously colonised individuals for ε. We use “leaky vaccine” only for σ and “leaky carriage immunity” only for ε throughout.*

**Figure 1:**
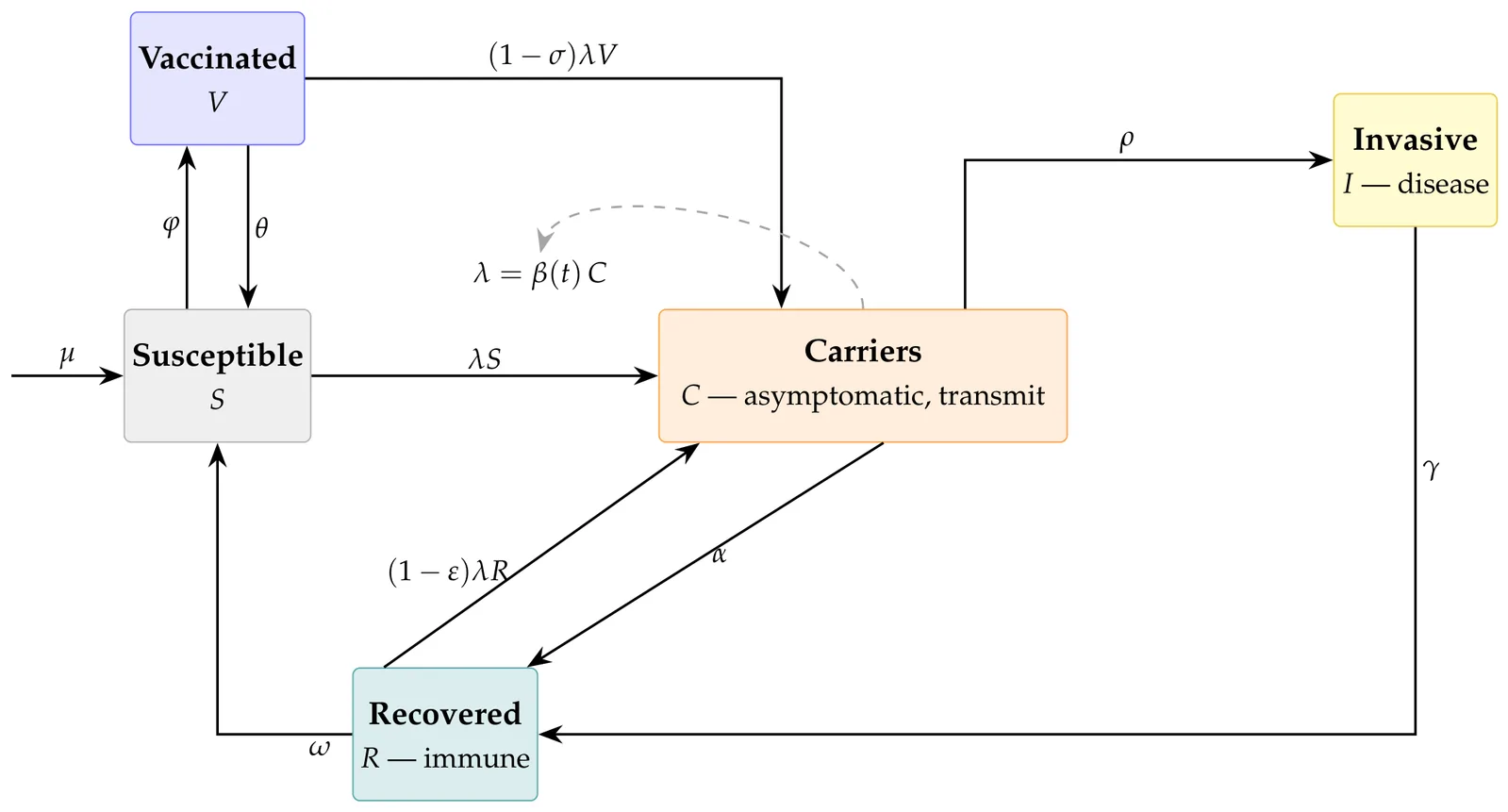
Compartmental structure of the carriage-based meningococcal model Equation (2). Carriers *C* are the only transmitting class; the dashed line is the force of infection λ=β(t)C, not a demographic flow. Susceptible and vaccinated individuals acquire carriage at rates λS and (1−σ)λV; the immune re-acquire at (1−ε)λR. Carriage clears to immunity at rate α or, rarely, progresses to invasive disease at rate ρ (ρ≪α). Invasive disease *I* drains only to *R* at rate γ; natural immunity wanes at ω and vaccine protection at θ. Mortality μ acts on every compartment and is drawn only at entry.

Carriage clears at rate α or, with small probability, proceeds to invasive disease at rate ρ. Since ρ/(ρ+α) is exactly the per-episode probability of progression to invasive disease, and case:carrier ratios reported for belt serogroups place this probability at $10^{-3}$–$10^{-2}$ [6,7] while carriage-duration studies give α≈4–$13\;{\mathrm{yr}}^{-1}$ (mean carriage duration of roughly 4–12 weeks), it follows that $\rho \approx \alpha \times (10^{-3}–10^{-2})\ll \alpha$. The compartmental structure is shown in Figure 1.

Individuals are susceptible (*S*), vaccinated (*V*), asymptomatic carriers (*C*), invasively diseased (*I*), or recovered and immune (*R*). Carriers are the sole transmitting class: susceptibles and vaccinees acquire carriage at rates λS and (1−σ)λV, and the immune re-acquire it at (1−ε)λR, where the force of infection λ=β(t)C (dashed) is driven by carriage prevalence, not by disease. Carriage either clears to immunity at rate α or, rarely, progresses to invasive disease at rate ρ; because ρ≪α, almost all carriage resolves without disease. Invasive disease is epidemiologically terminal, draining only to *R* at recovery rate γ and contributing nothing to onward transmission. Immunity wanes back to susceptibility at rate ω and vaccine protection at rate θ, while the mortality rate μ acts on every compartment. The governing system is

(2) $$\begin{array}{cc} \frac{dS}{dt} & =\mu +\omega R+\theta V-\lambda S-(\mu +\varphi )S,\\ \frac{dV}{dt} & =\varphi S-(1-\sigma )\lambda V-(\mu +\theta )V,\\ \frac{dC}{dt} & =\lambda \left[S+(1-\sigma )V+(1-\varepsilon )R\right]-\left(\rho +\alpha +\mu \right)C,\\ \frac{dI}{dt} & =\rho C-\left[\gamma +\mu +\delta (I)\right]I,\\ \frac{dR}{dt} & =\alpha C+\gamma I-(\omega +\mu )R-(1-\varepsilon )\lambda R, \end{array}$$

with all parameters positive and defined in Table 1. Here μ is the birth/death rate, φ the routine vaccination rate, θ the vaccine waning rate, ω the natural-immunity waning rate, and γ the recovery rate from invasive disease.

Case fatality is allowed to depend on health-system load through a capacity-saturating function

(3) $$\delta \left(I\right)=\delta_{T}\;\frac{K}{K+I}+\delta_{U}\;\frac{I}{K+I},$$

which interpolates between a treated fatality hazard $\delta_{T}$ (units ${\mathrm{yr}}^{-1}$) when case load *I* is small relative to capacity *K* and an untreated hazard $\delta_{U}>\delta_{T}$ when facilities are overwhelmed. We emphasise that $\delta_{T},\delta_{U}$ are hazards, not the case-fatality proportions usually reported in the clinical literature: with the invasive-disease compartment also clearing at rate γ (recovery) and μ (background mortality), the model’s implied case-fatality proportion at hazard δ is CFR=δ/(γ+μ+δ), so a reported CFR is converted to the hazard used in Equation (3) by δ=CFR(γ+μ)/(1−CFR); Table 1 reports both the source CFR and the resulting hazard for $\delta_{T},\delta_{U}$. We stress at the outset, and prove in Section 5, that $\delta_{U}$ and *K* affect burden but *not* the local bifurcation structure; $\delta_{T}$ and the recovery rate γ, by contrast, do affect the local bifurcation threshold $\varepsilon^{*}$, through the fraction of invasive cases that return to *R* (Remark 5), even though *I* remains an epidemiological dead end for transmission itself (it does not feed back into $R_{0}$ or into new carriage acquisition).

On the biologically relevant domain I≥0 (so K+I≥K>0), δ(·) is $C^{\infty }$ and hence locally Lipschitz, and the seasonal forcing β(t) of Equation (23) is continuous (indeed smooth) in *t*, so no Carathéodory-type argument is needed for it. The vector field of Equation (2) is therefore continuous in *t* and locally Lipschitz in (S,V,C,I,R), so the standard Picard–Lindelöf theorem gives a unique local solution through any initial condition, extended to all t≥0 by the a-priori bound established in Section 3. The one genuine discontinuity in this paper is not in the forcing but in the state trajectory itself: the instantaneous coverage pulse of Section 7 is a jump in (S,V,R) applied at a single instant, handled by restarting the (still Picard–Lindelöf-applicable) initial-value problem from the post-jump state, rather than by any weakening of the existence theory used elsewhere.

**Remark** **2.** *The term (1−ε)λR appearing in both the C and R equations is the modelling decision that distinguishes this system from a standard vaccination model: it states that individuals with protective immunity may still acquire and transmit carriage, grounded in the carriage-persistence evidence cited above [6,7]. As we show, this leaky re-acquisition term is the source of the model’s bistability.*

## 3. Basic Properties

Let $\Omega =\{\left(S,V,C,I,R\right)\in R_{+}^{5}:S+V+C+I+R\leq 1\}$.

**Proposition** **1** (Well-posedness)**.** *The region* Ω *is positively invariant for Equation* (2)*, and solutions with initial data in* Ω *remain non-negative and bounded for all t≥0.*

**Proof.**  On each face $x_{i}=0$ the corresponding derivative is non-negative (e.g., $\frac{dS}{dt}{|}_{S=0}=\mu +\omega R+\theta V\geq 0$), so the positive orthant is invariant. Summing Equation (2) with M=S+V+C+I+R gives $\frac{dM}{dt}=\mu -\mu M-\delta \left(I\right)I\leq \mu \left(1-M\right)$. Comparing M(t) with the solution $\bar{M}\left(t\right)$ of $\frac{d\bar{M}}{dt}=\mu \left(1-\bar{M}\right)$, $\bar{M}\left(0\right)=M\left(0\right)$, the standard comparison theorem for scalar ODEs gives $M\left(t\right)\leq \bar{M}\left(t\right)$ for all t≥0, and since $\bar{M}\left(t\right)=1+\left[M\left(0\right)-1\right]e^{-\mu t}$ solves the linear equation exactly,

(4) $$M\left(t\right)\;\leq \;1+\left[M(0)-1\right]e^{-\mu t},\;t\geq 0.$$

If M(0)≤1, the right-hand side of Equation (4) is ≤1 for every t≥0, since $e^{-\mu t}\in \left(0,1\right]$ and the bracket is ≤0; hence M(t)≤1 for all t≥0 pointwise. Thus Ω is positively invariant, and any trajectory started with M(0)>1 decays to Ω exponentially fast at rate μ. All solutions with initial data in Ω are therefore bounded.    □

## 4. The Disease-Free Equilibrium and the Reproduction Number

Setting C=I=0 in Equation (2) yields the disease-free equilibrium (DFE)

(5) $$E_{0}=\left(S^{0},V^{0},0,0,0\right),\;S^{0}=\frac{\mu +\theta }{\mu +\theta +\varphi },\;V^{0}=\frac{\varphi }{\mu +\theta +\varphi }.$$

We compute $R_{0}$ by the next-generation method [13]. The infected subsystem is (C,I). Because carriage produces disease but disease produces neither carriage nor further disease, the new-infection and transition matrices are

(6) $$F=\left(\begin{array}{cc} \beta_{0}\Phi & 0\\ 0 & 0 \end{array}\right),\;V=\left(\begin{array}{cc} K_{C} & 0\\ -\rho & d \end{array}\right),$$

where $\beta_{0}$ is the mean transmission rate, $K_{C}=\rho +\alpha +\mu$ is the total carriage exit rate, $d=\gamma +\mu +\delta_{T}$, and

(7) $$\Phi \;\equiv \;S^{0}+\left(1-\sigma \right)V^{0}=\frac{(\mu +\theta )+(1-\sigma )\varphi }{\mu +\theta +\varphi }$$

is the effective susceptibility of the carriage-acquiring pool. The construction $F_{22}=0$ encodes that invasive cases contribute zero new infections; this is supported, for the airborne community-level transmission this model targets, by the microbiological and epidemiological evidence discussed in the Introduction [6,7]. The spectral radius of $FV^{-1}$ is the (1,1) entry, giving the following.

**Proposition** **2** ($R_{0}$ is a carriage quantity)**.** *The basic reproduction number of Equation* (2) *is*

(8) $$R_{0}\;=\;\frac{\beta_{0}\;\Phi }{K_{C}}\;=\;\frac{\beta_{0}\left[(\mu +\theta )+(1-\sigma )\varphi \right]}{\left(\mu +\theta +\varphi )(\rho +\alpha +\mu \right)}.$$

*Because invasion is rare and carriage is short-lived, ρ≪α, so $K_{C}=\alpha +\mu +\rho \approx \alpha +\mu$ and $R_{0}\approx \beta_{0}\Phi /\left(\alpha +\mu \right)$. The invasion rate ρ enters $R_{0}$ only as a negligible perturbation to the carriage-clearance rate; the disease compartment I does not appear at all.*

**Remark** **3.** *Proposition 2 has a direct empirical consequence within this model structure. $R_{0}$ is determined by the duration of carriage and the contact rate, essentially independently of disease incidence. Treating notified invasive cases as though they were the transmitting population would therefore estimate a quantity distinct from the transmission potential that governs elimination; this is a different and weaker claim than asserting that case-incidence data are uninformative about carriage transmission in general. If invasive incidence is a stable, non-negative observation process proportional to carriage prevalence (as it is in this model, via ρC), its growth rate over an epidemic’s early phase can still be combined with an explicit observation model and generation-interval assumptions to recover information about the underlying carriage $R_{0}$; what is unsound is treating notified cases as the infectious population itself, not using them as surveillance data for a latent carriage process. Surveillance aimed directly at estimating $R_{0}$ should nonetheless target, or explicitly model, carriage, rather than treating notified disease counts as the transmitting compartment.*

**Remark** **4** (The basic reproduction number versus the effective reproduction number)**.** *$R_{0}$ as given by Equation* (8) *is, by construction, a property of the disease-free equilibrium $E_{0}$: it answers the question of whether a single carrier introduced into an otherwise completely naive population (no prior natural immunity, only whatever routine vaccination coverage φ is already running) will, on average, generate more than one secondary carrier. In a population where the pathogen has circulated before, a non-trivial fraction of individuals occupies the recovered/immune class R as well, and the quantity that governs whether prevalence is currently growing or shrinking is not $R_{0}$ itself but the* effective reproduction number

(9) $$R_{0}^{\mathrm{eff}}\left(t\right)\;=\;\frac{\beta \left(t\right)\left[S(t)+(1-\sigma )V(t)+(1-\varepsilon )R(t)\right]}{K_{C}},$$

*evaluated at the actual current state (S(t),V(t),R(t)) rather than at the disease-free values $\left(S^{0},V^{0},0\right)$; indeed Equation* (9) *is exactly the coefficient multiplying C in $\frac{dC}{dt}=K_{C}\left[R_{0}^{\mathrm{eff}}\left(t\right)-1\right]C$, so carriage prevalence is instantaneously growing iff $R_{0}^{\mathrm{eff}}\left(t\right)>1$. Because ε<1 discounts the contribution of the recovered class relative to what the same individuals would contribute as susceptibles, for any fixed total S(t)+V(t)+R(t) moving mass from S into R (as happens once the epidemic has run for a while and carriers have cleared into R) strictly decreases the bracketed sum, and hence $R_{0}^{\mathrm{eff}}\left(t\right)<R_{0}$ once R(t)>0, all else equal: accumulated natural immunity, like vaccination, depletes the effective susceptible pool below the disease-free level. The distinction matters operationally, not just formally: $R_{0}$ is a structural threshold parameter, estimable from the biological parameters in Table 1 without reference to any particular population’s exposure history, and it is what determines the critical vaccination coverage $\varphi_{c}$ in Equation* (10) *and the bifurcation structure of Section 5; $R_{0}^{\mathrm{eff}}\left(t\right)$ is a real-time, population-specific quantity that additionally requires knowing (or estimating, e.g., from seroprevalence surveys or documented vaccination coverage) the current partition of the population across S,V,R, and it is $R_{0}^{\mathrm{eff}}\left(t\right)$, not $R_{0}$, that should be tracked when assessing whether an ongoing outbreak in a specific district is waxing or waning. All numerical results in this manuscript (the bifurcation diagram, the seasonal attractor analysis, the vaccination scenarios) are computed from the full system Equation* (2) *and therefore already incorporate the time-varying state (S(t),V(t),R(t)) correctly; Equation* (9) *simply makes explicit, as a named quantity, the mechanism by which they do so, and gives programme evaluators a formula for the real-time indicator implied by the model.*

Setting $R_{0}=1$ and solving Equation (8) for φ gives the critical routine coverage

(10) $$\varphi_{c}=\frac{\left(\mu +\theta \right)\left(\beta_{0}-\alpha -\mu -\rho \right)}{\left(\rho +\alpha +\mu \right)-\beta_{0}\left(1-\sigma \right)},$$

valid when the denominator is positive; when $\beta_{0}\left(1-\sigma \right)\geq K_{C}$ the leaky vaccine cannot drive $R_{0}$ below unity at any coverage, a first indication that σ and ε control feasibility.

**Theorem** **1** (Local stability of the DFE)**.** *The disease-free equilibrium $E_{0}$ is locally asymptotically stable when $R_{0}<1$ and unstable when $R_{0}>1$.*

**Proof.**  The Jacobian of Equation (2) at $E_{0}$ is block triangular with respect to the ordering (C;S,V,I,R), because at C=I=0 no term in the S,V,I,R-equations depends on *C* except through λ, and *I* decouples from S,V,R entirely. The *C*-row decouples with eigenvalue $\beta_{0}\Phi -K_{C}=K_{C}\left(R_{0}-1\right)$, and the *I*-row decouples with eigenvalue −d, $d=\gamma +\mu +\delta_{T}$. The remaining 3×3 block, in the ordering (S,V,R), is

(11) $$A\;=\;\left(\begin{array}{ccc} -(\mu +\varphi ) & \theta & \omega \\ \varphi & -(\mu +\theta ) & 0\\ 0 & 0 & -(\mu +\omega ) \end{array}\right),$$

obtained from $\partial \left(\dot{S},\dot{V},\dot{R}\right)/\partial \left(S,V,R\right)$ at $E_{0}$ (the λ-terms vanish at C=I=0, so this block does not depend on Φ or $K_{C}$). Its characteristic polynomial is

(12) $$\begin{array}{cc} \det(\xi I-A) & \;=\;\left(\xi +\mu +\omega \right)\left[(\xi +\mu +\varphi )(\xi +\mu +\theta )-\varphi \theta \right]\\ \; & \;=\;\left(\xi +\mu +\omega \right)\left[\xi^{2}+\left(2\mu +\varphi +\theta \right)\xi +\mu \left(\mu +\varphi +\theta \right)\right], \end{array}$$

whose roots are ξ=−(μ+ω) together with the two roots of the quadratic factor $\xi^{2}+\left(2\mu +\varphi +\theta \right)\xi +\mu \left(\mu +\varphi +\theta \right)$. Direct expansion confirms $\left(\xi +\mu \right)\left(\xi +\mu +\varphi +\theta \right)=\xi^{2}+\left(2\mu +\varphi +\theta \right)\xi +\mu \left(\mu +\varphi +\theta \right)$, so the quadratic factors exactly as (ξ+μ)(ξ+μ+φ+θ), giving the full spectrum of *A* as

(13) −μ,−(μ+ω),−(μ+φ+θ),

all strictly negative for any positive parameters. The complete spectrum of the full Jacobian at $E_{0}$ is therefore

(14) $$K_{C}\left(R_{0}-1\right),\;-d,\;-\mu ,\;-\left(\mu +\omega \right),\;-\left(\mu +\varphi +\theta \right),$$

so every eigenvalue has negative real part iff $R_{0}<1$.    □

## 5. Backward Bifurcation

We now determine the direction of bifurcation at $R_{0}=1$ using the centre manifold theorem in the form of Castillo-Chavez and Song [14]. To avoid a clash of notation—the invasion rate and the Castillo-Chavez–Song coefficient are both conventionally denoted *a*—we write the invasion rate as ρ throughout and reserve a,b for the bifurcation coefficients. Let $\left(x_{1},\ldots ,x_{5}\right)=\left(S,V,C,I,R\right)$ and take β as the bifurcation parameter with critical value $\beta^{*}=K_{C}/\Phi$ (so that $R_{0}=1$).

At $\left(E_{0},\beta^{*}\right)$ the Jacobian has a simple zero eigenvalue. Writing $J_{0}$ for this Jacobian, the structure of the new-infection process is that new carriers are the only new infections, and they enter compartment $C=x_{3}$, forcing the left eigenvector to collapse onto $e_{3}$:

(15) v=(0,0,1,0,0).

Indeed, $vJ_{0}=0$ requires $v_{1}=v_{2}=v_{4}=v_{5}=0$ from columns 1,2,4,5, leaving $v_{3}$ free. The right eigenvector w solves $J_{0}w=0$ row by row. The *I*-row of Equation (2) gives, as before, $w_{4}=\rho /d$. The *R*-row, however, is $\frac{dR}{dt}=\alpha C+\gamma I-\left(\omega +\mu \right)R-\left(1-\varepsilon \right)\lambda R$: the linear term γI contributes γ to the (R,I) entry of $J_{0}$, so the *R*-row equation is $\alpha \;w_{3}+\gamma \;w_{4}-\left(\omega +\mu \right)w_{5}=0$, giving

(16) $$w_{4}=\frac{\rho }{d},\;w_{5}=\frac{\alpha^{\prime }}{\omega +\mu },\;\alpha^{\prime }\;:=\;\alpha +\gamma w_{4}\;=\;\alpha +\frac{\gamma \rho }{d},$$

where $\alpha^{\prime }$ is an *effective* carriage-clearance-to-*R* rate: the fraction ρ/d of carriers that invade returns to *R* at rate γ (rather than at rate α directly from *C*), so $\alpha^{\prime }$ augments α by this second route into *R*. Substituting $w_{5}=\alpha^{\prime }/\left(\omega +\mu \right)$ into the *S*- and *V*-rows, exactly as before, gives

(17) $$w_{1}=-\frac{(\mu +\theta )P+\theta Q}{D_{2}},\;w_{2}=-\frac{(\mu +\varphi )Q+\varphi P}{D_{2}},$$

where $D_{2}=\mu \left(\mu +\theta +\varphi \right)>0$, $P=\beta^{*}S^{0}-\omega \alpha^{\prime }/\left(\omega +\mu \right)$, and $Q=\left(1-\sigma \right)\beta^{*}V^{0}$—the same formulas as before, but with α replaced throughout by $\alpha^{\prime }$.

**Remark** **5** (Correction: $w_{5}$ omitted the γI return flow)**.** *An earlier version of this derivation set $w_{5}=\alpha /\left(\omega +\mu \right)$, overlooking the γ entry that $\frac{dR}{dt}=\alpha C+\gamma I-\cdots$ contributes to the (R,I) position of $J_{0}$. Because $w_{4}=\rho /d\neq 0$, this entry is not negligible: it was caught on further review and is corrected throughout this section as $w_{5}=\alpha^{\prime }/\left(\omega +\mu \right)$ with $\alpha^{\prime }=\alpha +\gamma \rho /d$ above. The correction changes $\varepsilon^{*}$ quantitatively (see Section Backward Bifurcation Requires Vaccination) but, as we show below, leaves Corollary 2 (no backward bifurcation without vaccination) intact, since $K_{C}-\alpha^{\prime }>0$ always.*

Because $v=e_{3}$, only the second derivatives of the third component $f_{3}=\lambda \left[S+\left(1-\sigma \right)V+\left(1-\varepsilon \right)R\right]-K_{C}C$ survive. The non-zero second-order partials at $E_{0}$ are the bilinear terms $\partial^{2}f_{3}/\partial x_{1}\partial x_{3}=\beta^{*}$, $\partial^{2}f_{3}/\partial x_{2}\partial x_{3}=\left(1-\sigma \right)\beta^{*}$, and $\partial^{2}f_{3}/\partial x_{5}\partial x_{3}=\left(1-\varepsilon \right)\beta^{*}$. The Castillo-Chavez–Song coefficients therefore reduce to

(18) $$\begin{array}{cc} a & =\sum_{k,i,j}v_{k}\;w_{i}w_{j}\;\frac{\partial^{2}f_{k}}{\partial x_{i}\partial x_{j}}{|}_{E_{0}}=2\beta^{*}\left[w_{1}+\left(1-\sigma \right)w_{2}+\left(1-\varepsilon \right)w_{5}\right], \end{array}$$

(19) $$\begin{array}{cc} b & =\sum_{k,i}v_{k}\;w_{i}\;\frac{\partial^{2}f_{k}}{\partial x_{i}\partial \beta }{|}_{E_{0}}=\Phi =S^{0}+\left(1-\sigma \right)V^{0}\;>\;0. \end{array}$$

Since b>0 unconditionally, the direction of bifurcation is governed entirely by the sign of *a*.

**Figure 2:**
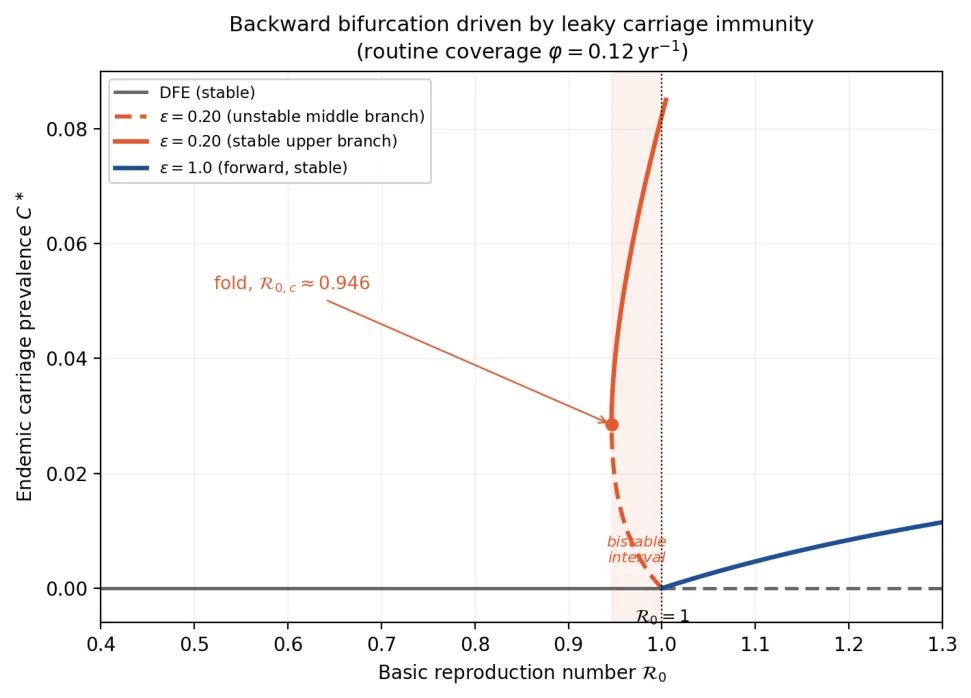
Backward bifurcation driven by leaky carriage immunity under routine vaccination coverage $\varphi =0.12\;{\mathrm{yr}}^{-1}$ (Corollary 2: no fold exists at φ=0). Endemic carriage prevalence $C^{*}$ against $R_{0}$. Solid curves: stable branches (upper endemic branch and the DFE branch); dashed curves: unstable branch, including the unstable middle branch between the fold at $R_{0,c}\approx 0.946$ and $R_{0}=1$. For leaky immunity (ε=0.20, coral) the branch folds back to $R_{0,c}\approx 0.946<1$, the signature of backward bifurcation, with the bistable interval $\left[R_{0,c},1\right)$ shaded; for solid immunity (ε=1, blue) the bifurcation is forward and no fold occurs. Other parameters as in Table 1.

By the Castillo-Chavez–Song theorem, this calculation is a strictly *local* result: it determines the sign of the leading coefficient in the reduced dynamics on the one-dimensional centre manifold in a neighbourhood of $\left(E_{0},\beta^{*}\right)$, and hence the direction in which the emerging branch of non-trivial equilibria bends at $R_{0}=1$. It does not, by itself, establish the global extent of a bistable region or the stability of endemic equilibria away from $R_{0}=1$; the global bistable structure reported in Figure 2 below is established separately, by numerical equilibrium continuation.

**Theorem** **2** (Backward-bifurcation condition)**.** *System Equation* (2) *undergoes a backward bifurcation at $R_{0}=1$ if and only if a>0, that is, if and only if*

(20) $$\frac{\left(1-\varepsilon \right)\;\alpha^{\prime }\;\mu \;\left(\mu +\theta +\varphi \right)}{\omega +\mu }\;>\;P\;\Pi +Q\;\Theta ,$$

*where $\alpha^{\prime }=\alpha +\gamma \rho /d$ as in Equation* (16)*, Π=(μ+θ)+(1−σ)φ, and Θ=θ+(1−σ)(μ+φ). Equivalently, defining the carriage-immunity threshold*

(21) $$\varepsilon^{*}=1-\frac{\left(P\Pi +Q\Theta )(\omega +\mu \right)}{\alpha^{\prime }\;\mu \;\left(\mu +\theta +\varphi \right)},$$

*the bifurcation at $R_{0}=1$ is backward precisely when $\varepsilon <\varepsilon^{*}$.*

**Proof.**  By Castillo-Chavez and Song [14] (Theorem 4.1), with b>0 the bifurcation is backward iff a>0. Substituting Equations (16) and (17) into Equation (18) and clearing the positive factor $2\beta^{*}/D_{2}$ yields $\left(1-\varepsilon \right)w_{5}D_{2}>\left(\mu +\theta \right)P+\theta Q+\left(1-\sigma \right)\left[\left(\mu +\varphi \right)Q+\varphi P\right]$. Collecting the *P* and *Q* terms gives PΠ+QΘ on the right and, with $w_{5}=\alpha^{\prime }/\left(\omega +\mu \right)$ and $D_{2}=\mu \left(\mu +\theta +\varphi \right)$, the left-hand side of Equation (20). Because ε enters only the left-hand side and linearly, solving the inequality for ε gives Equation (21).    □

**Corollary** **1** (Which case-management parameters can shift the bifurcation)**.** *Unlike the invasion rate ρ—already known to enter implicitly through $\beta^{*}$—the recovery rate γ and the treated case-fatality rate $\delta_{T}$ enter the bifurcation condition Equation* (20) *as well, through $\alpha^{\prime }=\alpha +\gamma \rho /d$ with $d=\gamma +\mu +\delta_{T}$ (Remark 5): a larger γ (faster recovery from invasive disease) or smaller $\delta_{T}$ (more effective treatment) both increase $d^{-1}$-weighted return flow into R and hence shift $\varepsilon^{*}$. This corrects an earlier version of this corollary, which claimed γ and $\delta_{T}$ were absent; that claim rested on the uncorrected $w_{5}=\alpha /\left(\omega +\mu \right)$ of Remark 5 and is withdrawn.*

*What remains true, and follows from the same local linearisation at I=0, is narrower: $\delta_{U}$ and K—the untreated fatality rate and the capacity parameter of Equation* (3)*—do not appear anywhere in a, b, P, Q, $\beta^{*}$, $\alpha^{\prime }$, or $\varepsilon^{*}$. Both enter δ(I) only through the combination $\delta_{U}I/\left(K+I\right)$, whose derivative with respect to I vanishes at I=0 (the disease-free equilibrium); consequently $\delta \left(0\right)=\delta_{T}$ regardless of $\delta_{U}$ and K, and the local bifurcation calculation, which linearises at I=0, cannot see either parameter. Health-system capacity and untreated case fatality therefore still cannot create, remove, or shift this* local *bifurcation; they affect disease burden (via Section 7) but not the local direction of bifurcation at $R_{0}=1$. Treatment speed (γ) and treated-case fatality ($\delta_{T}$) are a different matter, and do now enter, as corrected above. Unlike $\delta_{U}$ and K, ρ also enters twice over—both through $\alpha^{\prime }$ directly, and (as previously noted) implicitly through $\beta^{*}=K_{C}/\Phi =\left(\rho +\alpha +\mu \right)/\Phi$ and hence through P and Q—so of the five post-carriage parameters $\left(\rho ,\gamma ,\delta_{T},\delta_{U},K\right)$, only $\delta_{U}$ and K remain provably inert to the local bifurcation structure.*

Corollary 1 is a partial, corrected structural counterpart of Proposition 2: *all* post-carriage parameters $\left(\rho ,\gamma ,\delta_{T},\delta_{U},K\right)$ are absent from $R_{0}=\beta \Phi /K_{C}$ (since $K_{C}=\rho +\alpha +\mu$ and none of $\gamma ,\delta_{T},\delta_{U},K$ appear in it), but only $\delta_{U}$ and *K* are also absent from the local bifurcation condition Equation (20); $\rho ,\gamma ,\delta_{T}$ affect where the bifurcation sits (via $\alpha^{\prime }$ and $\beta^{*}$) even though they do not affect whether $R_{0}=1$ is crossed. A “health-system capacity drives bistability” argument therefore still fails here for capacity *K* and untreated fatality $\delta_{U}$ specifically, but treatment speed and treated-case fatality are not, after correction, in the same inert category.

**Remark** **6** (What actually drives bistability)**.** *Inspection of Equation* (20) *identifies three channels. (i)* Susceptible depletion*, the PΠ and QΘ terms, opposes backward bifurcation* when P,Q≥0*; $Q=\left(1-\sigma \right)\beta^{*}V^{0}\geq 0$ always, but $P=\beta^{*}S^{0}-\omega \alpha^{\prime }/\left(\omega +\mu \right)$ is a difference of two positive quantities and is not sign-definite. P>0 holds when $\beta^{*}S^{0}\left(\omega +\mu \right)>\omega \alpha^{\prime }$; at φ=0 (Corollary 2 below) P≥ρ+μ>0 for every ω≥0 (shown in the proof of Corollary 2), so susceptible depletion is stabilising throughout in that case, but for φ>0, P can turn negative for sufficiently large ω. (ii)* Leaky carriage immunity*, ε<1, supplies the driving term on the left of Equation* (20)*. When P>0 (susceptible depletion stabilising), setting ε=1 forces a<0: leaky immunity is then* required *for backward bifurcation, since solid immunity alone cannot produce it in that regime. When P<0 instead, this requirement drops away: backward bifurcation can arise even at ε=1, driven purely by fast natural-immunity waning outrunning carriage clearance—a distinct, second mechanism from leaky carriage immunity itself. (iii)* Strong immunity waning *therefore enters non-monotonically: ω drives P downward (favouring backward bifurcation once P<0) but also appears in the denominator prefactor (ω+μ) of Equation* (21) *and in $w_{5}=\alpha^{\prime }/\left(\omega +\mu \right)$ (favouring forward bifurcation as ω grows, since $w_{5}$ shrinks). The net effect is resolved numerically in Section 7.*

### Backward Bifurcation Requires Vaccination

A furtherstructural feature of Equation (20) is worth isolating explicitly, since it determines where the bifurcation shown in Figure 2 can and cannot be observed.

**Corollary** **2** (Backward bifurcation is impossible without vaccination)**.** *At φ=0, $\varepsilon^{*}<0$ for every choice of the remaining positive parameters $\alpha ,\gamma ,\delta_{T},\mu ,\omega ,\rho ,\theta$; consequently system Equation* (2) *cannot undergo backward bifurcation at $R_{0}=1$ when φ=0, for any admissible parameter values and any ε∈[0,1].*

**Proof.**  At φ=0 the vaccinated class is empty, $V^{0}=0$, so $\Phi =S^{0}=1$ and $\beta^{*}=K_{C}=\rho +\alpha +\mu$ identically, and Q=0. Substituting into Equation (21) with Π=μ+θ, QΘ=0, and simplifying,

(22) $$\begin{array}{cc} \varepsilon^{*}\; & =1-\frac{P(\omega +\mu )}{\alpha^{\prime }\mu },\;P=K_{C}-\frac{\omega \alpha^{\prime }}{\omega +\mu },\\ K_{C}-\alpha^{\prime } & =\rho +\mu -\frac{\gamma \rho }{d}=\frac{\rho (\delta_{T}+\mu )}{d}+\mu \;>\;0, \end{array}$$

using $\alpha^{\prime }=\alpha +\gamma \rho /d$ (Remark 5) and $d=\gamma +\mu +\delta_{T}$. Because $K_{C}-\alpha^{\prime }>0$ strictly (the displayed identity is a sum of positive terms for any positive $\rho ,\mu ,\delta_{T},d$), the same argument as before goes through with $\alpha^{\prime }$ in place of α: expanding, $\varepsilon^{*}=-\left[\omega \left(K_{C}-\alpha^{\prime }\right)/\mu +\left(K_{C}-\alpha^{\prime }\right)\right]/\alpha^{\prime }$, a strictly negative quantity for every ω≥0 and every positive $\rho ,\mu ,\gamma ,\delta_{T},\theta$. Since $\varepsilon \geq 0>\varepsilon^{*}$ always, Theorem 2 gives a<0 (forward bifurcation) at φ=0 regardless of parameter values. The correction to $w_{5}$ (Remark 5) thus changes the numerical value of $\varepsilon^{*}$ at φ>0 (Section Backward Bifurcation Requires Vaccination below) but leaves this corollary intact, because $K_{C}>\alpha^{\prime }$ is preserved by the same margin argument that gave $K_{C}>\alpha$ before.    □

Backward bifurcation therefore only becomes possible once routine vaccination coverage is introduced. Increasing φ increases $\beta^{*}=K_{C}/\Phi$ (since Φ<1 for φ>0, σ>0), which can drive *P* negative for moderate φ, opening a window in which $\varepsilon^{*}>0$. At the routine coverage used elsewhere in this manuscript, $\varphi =0.12\;{\mathrm{yr}}^{-1}$ (Section 7), Equation (21) gives $\varepsilon^{*}\approx 0.386$ at the remaining baseline parameters of Table 1 (using the corrected $\alpha^{\prime }$ of Remark 5 and the fatality hazards of Table 1)—comfortably inside [0,1] and below the baseline ε=0.55 (see Section 7 for what this means for Figure 2). More generally, at these baseline parameters $\varepsilon^{*}$ crosses zero at φ≈0.0074 and increases monotonically with φ thereafter, peaking around $\varepsilon^{*}\approx 0.60$ near φ≈1.2 and then easing only slightly as φ→∞ ($\varepsilon^{*}\approx 0.60$ still at φ=3); it therefore remains within the admissible interval (0,1) for every routine coverage above this small threshold, all the way through and beyond the plausible programmatic range, rather than only at the specific value used in Section 7 or over some bounded “intermediate” band. (An earlier version of this paragraph additionally claimed that $\varepsilon^{*}$ falls back toward zero at large φ, confining the effect to an intermediate range of coverage; this was inconsistent with the plateau just described and is withdrawn—the QΘ term grows with φ but never overtakes the driving term enough to close the window within the admissible range.) Backward bifurcation in this model is thus not a property of the unvaccinated carriage dynamics but a vaccination-induced phenomenon, of the same family identified for other partially effective vaccines by Kribs-Zaleta and Velasco-Hernández [15]: a vaccine that is imperfect against carriage acquisition can, through its effect on the susceptible pool, create a bistable region that would not exist in its absence.

Figure 2 confirms this analysis numerically. We computed the branch by full numerical equilibrium continuation of Equation (2), with φ=0.12 fixed and β (equivalently $R_{0}=\beta \Phi /K_{C}$) as the free parameter, parametrising the branch by carriage prevalence *C* rather than by $R_{0}$ so that the continuation passes smoothly through the fold (standard practice, since $C(R_{0})$ is multi-valued near a fold while $R_{0}\left(C\right)$ is not): for each *C* on a fine grid we solved the remaining four equilibrium equations for (β,S,V,I,R) by Newton’s method with an analytically computed Jacobian, using the solution at the previous *C* as the initial guess. Stability at each computed point was determined from the sign of the real part of the eigenvalues of the full 5×5 Jacobian of Equation (2) evaluated at that equilibrium—direct linearisation, not the local centre-manifold coefficient of Theorem 2. The fold was located as the point of minimum $R_{0}$ along the continued branch, at $R_{0,c}\approx 0.946$ (using the corrected fatality hazards of Table 1); for ε=1 the same continuation, parametrised directly by $R_{0}$ since no fold arises, is monotonic and stable throughout, with no turning point up to $R_{0}=1.8$. With leaky immunity ($\varepsilon =0.20<\varepsilon^{*}\approx 0.386$) the endemic branch folds back to $R_{0,c}\approx 0.946$, producing a region of bistability between $R_{0,c}$ and 1 in which a stable endemic state coexists with the stable DFE; with solid immunity (ε=1) the bifurcation is forward and $R_{0}<1$ guarantees elimination. We stress that ε=0.20 is the *lower bound* of the plausible range in Table 1, not the baseline value ε=0.55: at baseline, $\varepsilon =0.55>\varepsilon^{*}\approx 0.386$, so the bifurcation at φ=0.12 is *forward* and Figure 2 does not depict the baseline parameterisation. Repeating the continuation at ε=0.333 and ε=0.36—both still below baseline but closer to it—still produces a fold, at $R_{0,c}\approx 0.992$ and $R_{0,c}\approx 0.998$ respectively; the fold disappears only once ε approaches $\varepsilon^{*}\approx 0.386$ from below. Bistability at φ=0.12 is therefore confined to the lower part of the plausible ε range, and the policy statements below about a catch-up campaign being “necessary rather than merely helpful” (Section 7) should be read as applying to that lower part of the range, not to the baseline parameterisation.

## 6. Seasonal Forcing and the Search for Recurrence

We now let the transmission rate vary seasonally,

(23) $$\beta \left(t\right)=\beta_{0}\left[1+\beta_{1}\cos\left(2\pi t+\psi \right)\right],\;0\leq \beta_{1}<1,$$

with *t* in years, and ask whether the multi-annual recurrence hypothesised for the belt can arise from annual forcing and immunity waning alone, within this model.

The reproduction number of the forced system.

For a periodic environment the relevant threshold quantity in general is the basic reproduction number of the periodic system in the sense of Bacaër and Guernaoui [16], defined as the spectral radius of the next-infection operator on the space of 1-periodic functions. Because the carriage subsystem is scalar, this operator reduces to the integral kernel $K\left(t,s\right)=\beta \left(t\right)\Phi \;e^{-K_{C}\left(t-s\right)}$, and stability of the disease-free periodic state is determined by the Floquet exponent of the scalar carriage equation linearised at $E_{0}$,

(24) $$\Lambda =\int_{0}^{1}\left[\beta \left(t\right)\Phi -K_{C}\right]\;dt=\Phi \langle \beta \rangle -K_{C}=K_{C}\left(R_{0}-1\right),$$

where $R_{0}=\Phi \beta_{0}/K_{C}$ is exactly the autonomous reproduction number of Proposition 2 evaluated at the mean transmission rate $\beta_{0}$, since $\langle \beta \rangle =\beta_{0}$ exactly for the sinusoidal forcing Equation (23) ($\int_{0}^{1}\cos\left(2\pi t+\psi \right)\;dt=0$ for any phase ψ). For this scalar, exponentially clearing-carriage linearisation, the periodic reproduction number and the naive time-average threshold therefore coincide exactly, because the next-generation operator reduces to a convolution against an exponential kernel that integrates the forcing linearly; this need not hold in general periodic epidemic models with non-exponential infectious-period distributions or with feedback through more than one infected compartment, but no separate periodic-threshold calculation is required for the present model. The DFE periodic orbit is stable iff $R_{0}<1$, consistent with Theorem 1.

Attractor structure.

Beyond threshold, the interaction of annual forcing with the immunity timescale 1/ω can in principle generate a hierarchy of attractors through period-doubling and subharmonic resonance, as in other seasonally forced epidemic models. We investigated this computationally rather than relying on the linear intuition that a slow second timescale is sufficient for such resonance.

We integrated Equation (2) under Equation (23) (baseline parameters of Table 1, φ=0, ε=0.55, $\beta_{0}=8.29\;{\mathrm{yr}}^{-1}$ giving mean $R_{0}\approx 1.35$) with an adaptive-step embedded Runge–Kutta (4,5) scheme (absolute/relative tolerance $10^{-11}$/$10^{-8}$), discarded a transient, and sampled carriage prevalence *C* once per year (the annual stroboscopic map). A trajectory was classified as period-*p* if $\max_{n}\left|C\left(n+p\right)-C\left(n\right)\right|$, over the retained stroboscopic samples, was below 1% of the mean sampled value, for the smallest such p∈{1,…,8}; trajectories for which no such *p* was found were re-examined with a longer discarded transient and sampling window before any conclusion of genuine irregularity, since we found that apparent irregularity at the slowest waning rates tested was in every case a transient artefact rather than an attractor property.

**Figure 3:**
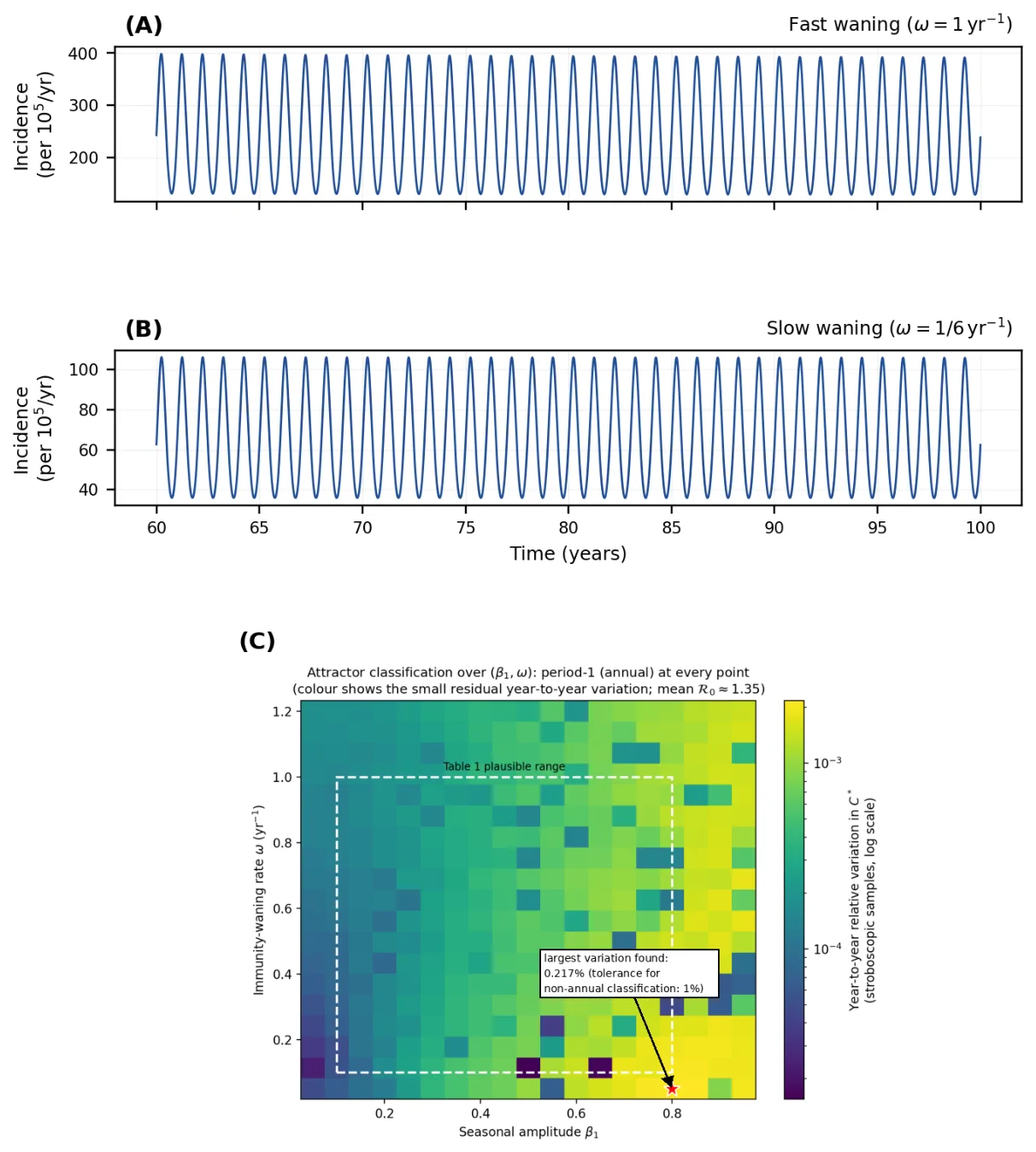
Seasonally forced dynamics show only annual (period-1) recurrence, at a representative pair of regimes and across the full swept parameter range. (**A**,**B**) Simulated invasive incidence, years 60–100 of a 100-year run (transient discarded), at identical mean transmissibility ($R_{0}\approx 1.35$) and seasonal amplitude ($\beta_{1}=0.55$): (**A**) fast immunity waning ($\omega =1\;{\mathrm{yr}}^{-1}$); (**B**) slow waning ($\omega =1/6\;{\mathrm{yr}}^{-1}$). Only the immunity timescale differs between the two; both settle to a period-1 (annual) stroboscopic map, verified out to 500 years. (**C**) Attractor classification over seasonal amplitude $\beta_{1}$ and immunity-waning rate ω (19×19 grid, 220-year integrations, first 150 years discarded). Colour shows the year-to-year relative variation in stroboscopically sampled carriage prevalence $C^{*}$ at each grid point (log scale); every point classifies as period-1 (annual) under the 1% tolerance used throughout (periods 1–8 tested), with the largest variation found, 0.217% (starred), still more than 4.6-fold below that tolerance and plausibly attributable to solver/transient noise at the $\mathrm{rtol}=10^{-8}$ level used for these long integrations (see text). The dashed box marks the range of $\beta_{1}$ and ω stated as plausible in Table 1. No multi-annual or irregular region was found anywhere in the swept domain.

Figure 3A,B contrasts two regimes at identical mean transmissibility ($R_{0}\approx 1.35$) and seasonal amplitude ($\beta_{1}=0.55$): fast immunity waning ($\omega =1\;{\mathrm{yr}}^{-1}$) and slow waning ($\omega =1/6\;{\mathrm{yr}}^{-1}$). Both converge to a simple annual (period-1) cycle; we verified this is a genuine converged attractor, not a slow transient, by re-running to 500 years and confirming the stroboscopic samples over the final 100 years still vary by <0.1%.

Figure 3C extends this to a full grid over $\beta_{1}\in \left[0.05,0.95\right]$ and $\omega \in \left[0.05,1.2\right]\;{\mathrm{yr}}^{-1}$—a range slightly wider than Table 1’s stated bounds, to check for a transition boundary just outside them—using 19×19 grid points, 220-year integrations with the first 150 years discarded (a longer transient than the representative time series of Figure 3A,B, needed because convergence is slower at the smallest ω tested). Every grid point classified as period-1; Figure 3C displays the underlying year-to-year relative variation in $C^{*}$ at each grid point (rather than the binary classification alone, which would carry no visual information once every point receives the same label), confirming that the grid was in fact computed pointwise. This residual variation is always at least 4.6-fold below the 1% classification tolerance (largest value 0.217%) and sits at the $10^{-4}$–$10^{-3}$ level against an integration tolerance of $\mathrm{rtol}=10^{-8}$; at that scale it is not distinguishable from solver or residual-transient noise, and we do not read its mild variation across the grid as a systematic approach to resonance. We also tested, outside the grid, higher $R_{0}$ (up to 5), longer carriage duration (up to 365 days, versus the baseline 60), and forcing directly across the bistable window identified in Section Backward Bifurcation Requires Vaccination (φ=0.12, ε=0.20, mean $R_{0}$ straddling $R_{0,c}\approx 0.946$–1); none produced anything other than convergence to a period-1 cycle, or, below the effective threshold, decay to the DFE.

We are not able, within this deterministic, single-strain, homogeneously mixing model, to reproduce multi-annual recurrence from seasonal forcing and immunity waning alone, at any parameter combination we tried. The fast relaxation of carriage ($1/K_{C}≲1$ year even at the longest carriage duration tested) is not by itself an adequate explanation; however, *S* and *R*, not *C*, are the slow variables in this model, and forced models of SIR type with a short infectious period are well known to support subharmonic resonance driven by slow susceptible/immune-class dynamics interacting with annual forcing (e.g., measles- and pertussis-type models). To characterise this more precisely, we linearised Equation (2) at the unforced ($\beta_{1}=0$) endemic equilibrium at baseline parameters and computed the Jacobian eigenvalues as the waning rate ω was varied from 1 down to $0.05\;{\mathrm{yr}}^{-1}$. In every case the leading pair is a complex–conjugate pair with intrinsic period 2π/|Im(λ)|≈5.5 years at ω=1, lengthening to ≈16.5 years at ω=0.05; overlapping the belt’s observed 5–12-year interval throughout most of this range, but with e-folding decay time −1/Re(λ) ranging from about 1.1 years (fast waning) to about 12.8 years (slow waning); Table 2 reports the full set. The model therefore does contain a damped oscillatory mode at approximately the right intrinsic timescale, but it decays under purely deterministic annual forcing rather than being sustained or amplified by it. This points toward stochastic amplification of these damped cycles environmental or demographic noise repeatedly re-exciting a mode that the deterministic skeleton alone lets decay, a mechanism well documented for measles and other recurrent-epidemic systems [17], as the most natural next step for reproducing the belt’s irregular multi-annual pattern, rather than a further search over this deterministic model’s parameter space.

We also compared this model directly against the structure used by Irving et al. [9], whose simulations do produce multi-annual recurrence under seasonal forcing and waning immunity. The key structural difference is that Irving et al. include an explicit, slowly waning serogroup-specific immune compartment interacting with strain replacement/competition, giving the forced system an additional slow variable and a nonlinear strain-interaction term that this single-strain, single-immunity-class model does not have; our model’s single carriage-immunity class *R* plays a broadly analogous slow-variable role but without the multi-strain replacement dynamics that, in their model, couples to it. We regard supplying that missing ingredient; either multi-strain structure or stochastic amplification of the damped mode identified above, or both together as the two most promising extensions for future work, rather than as evidence that annual forcing and waning immunity are individually irrelevant to the belt’s recurrence pattern.

## 7. Vaccination Scenarios and Burden

The model is age-homogeneous, so φS vaccinates susceptible individuals of all ages continuously at rate φ; setting $\varphi =0.12\;{\mathrm{yr}}^{-1}$ is therefore not a representation of a vaccine administered specifically to a 9–18-month birth cohort, and an instantaneous reduction of S+R is not a representation of coverage restricted to a specific age band. We treat the two interventions below as useful abstract analogues—a continuous, non-age-targeted uptake process versus an instantaneous pulse affecting a fixed fraction of the non-carrier, non-vaccinated population—calibrated in magnitude, not in age-targeting, to the corresponding WHO SAGE recommendations [5]; a genuinely age-targeted evaluation would require an age-stratified extension of the model, which we return to in Section 8.

We evaluate four programmatic scenarios against a no-vaccination baseline, holding the transmissibility at $\beta_{0}=8.29\;{\mathrm{yr}}^{-1}$ (as in Section 6) and forcing seasonally with $\beta_{1}=0.5$, phase ψ=0 (peak transmission at t≡0(mod1)): (1) no vaccination; (2) continuous routine uptake at rate $\varphi =0.12\;{\mathrm{yr}}^{-1}$, loosely parameterised in magnitude to the SAGE-recommended 9–18-month schedule [5]; (3) routine uptake plus a one-off, instantaneous coverage pulse at t=0 (coincident with the start of routine coverage) reaching 60% of susceptible and recovered individuals (moved into *V*), loosely parameterised in magnitude to the recommended 1–19-year catch-up [5]; and (4) routine uptake with a higher carriage efficacy (σ=0.85). All four scenarios share the same initial condition at t=0: the endemic seasonal state reached from a 30-year unvaccinated (φ=0) burn-in started near the disease-free state ($S\left(0\right)=1-10^{-4}$, $C\left(0\right)=10^{-4}$, all other compartments zero), so that scenario differences reflect only the intervention applied from t=0 onward and not an arbitrary initial transient; this burn-in state is $\left(S,V,C,I,R\right)\approx \left(0.560,0,0.0239,2.7\times 10^{-5},0.413\right)$. All four ODE systems were integrated with scipy.integrate.solve_ivp (LSODA, rtol $=10^{-9}$, atol$=10^{-12}$, maximum step 0.05yr) over 25 years; code reproducing this section, including the burn-in, is included in the supplementary notebook.

Cumulative invasive incidence is defined as ∫ρCdt (new entries to the *I* compartment, i.e., notified-case-equivalent invasive episodes), reported per $10^{5}$ of the normalised population N=1; disease-induced mortality δ(I)I leaves the population M=S+V+C+I+R slightly below 1 over the simulated horizon, and cumulative incidence and deaths are both reported relative to the fixed reference $10^{5}$, not to the (very slightly depleted) instantaneous population, so that the two remain directly comparable across scenarios. Cumulative invasive incidence and deaths over a 25-year horizon are computed with case fatality following Equation (3) at treatment capacity K=50 per $10^{5}$.

**Figure 4:**
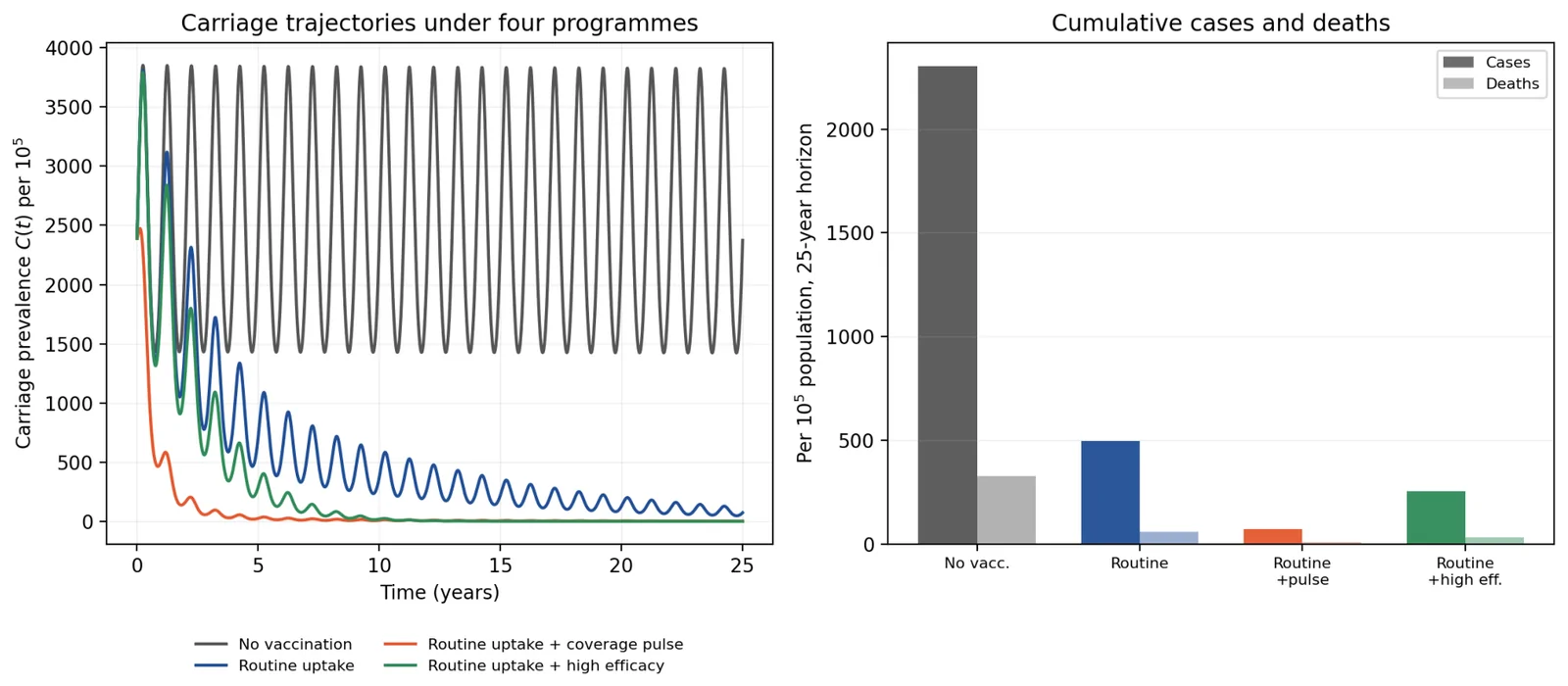
(**Left**) Carriage-prevalence trajectories under the four programmes, starting from the shared endemic seasonal state described in the text. (**Right**) Cumulative cases (solid) and deaths (faded) per $10^{5}$ over 25 years. Routine uptake alone reduces cumulative 25-year cases by about 78% relative to baseline (496.3 vs. 2302.3 per $10^{5}$; Table 3). Adding a coverage pulse, which transiently pushes the carriage reservoir below threshold, is far more effective than routine uptake alone (a 97% reduction); higher carriage efficacy improves on routine uptake without a pulse.

The results in Figure 4 and Table 3 show three things. Routine uptake alone reduces 25-year burden by about 78% relative to baseline but, within this 25-year horizon, does not eliminate carriage: the reservoir declines but has not reached its long-run level by year 25, because routine uptake protects new entrants slowly rather than clearing existing carriers. This is a statement about the timescale of decline, not about the ultimate outcome: at φ=0.12 and our baseline immunity ε=0.55, mean transmission gives $R_{0}\approx 0.98<1$ (using $\beta_{0}=8.29$), and because ε=0.55 lies above $\varepsilon^{*}\approx 0.386$ (Section Backward Bifurcation Requires Vaccination) the disease-free state is, at these baseline parameters, the only locally stable outcome; extending the routine-uptake simulation to 200 years shows carriage falling to $O(10^{-12})$, i.e., routine uptake alone does eventually eliminate at baseline parameters, just much more slowly than a 25-year programme horizon shows. A one-off coverage pulse, by contrast, transiently drives carriage prevalence below the transmission threshold and collapses cumulative burden by a further order of magnitude within the 25-year horizon—the numerical expression of the fact that carriage, not disease, is the reservoir, and that a pulse addresses the slow-decline problem directly by clearing existing carriers rather than only slowing new acquisition. Improving carriage efficacy σ substitutes partially for a pulse. The bistability discussed in Section Backward Bifurcation Requires Vaccination is a distinct phenomenon from this baseline scenario: it arises only in the lower part of the plausible ε range (below about 0.386 at φ=0.12), where—unlike at our baseline ε=0.55 —a stable endemic state coexists with the stable disease-free state and a pulse is needed not merely to accelerate decline but to escape the endemic branch entirely.

**Figure 5:**
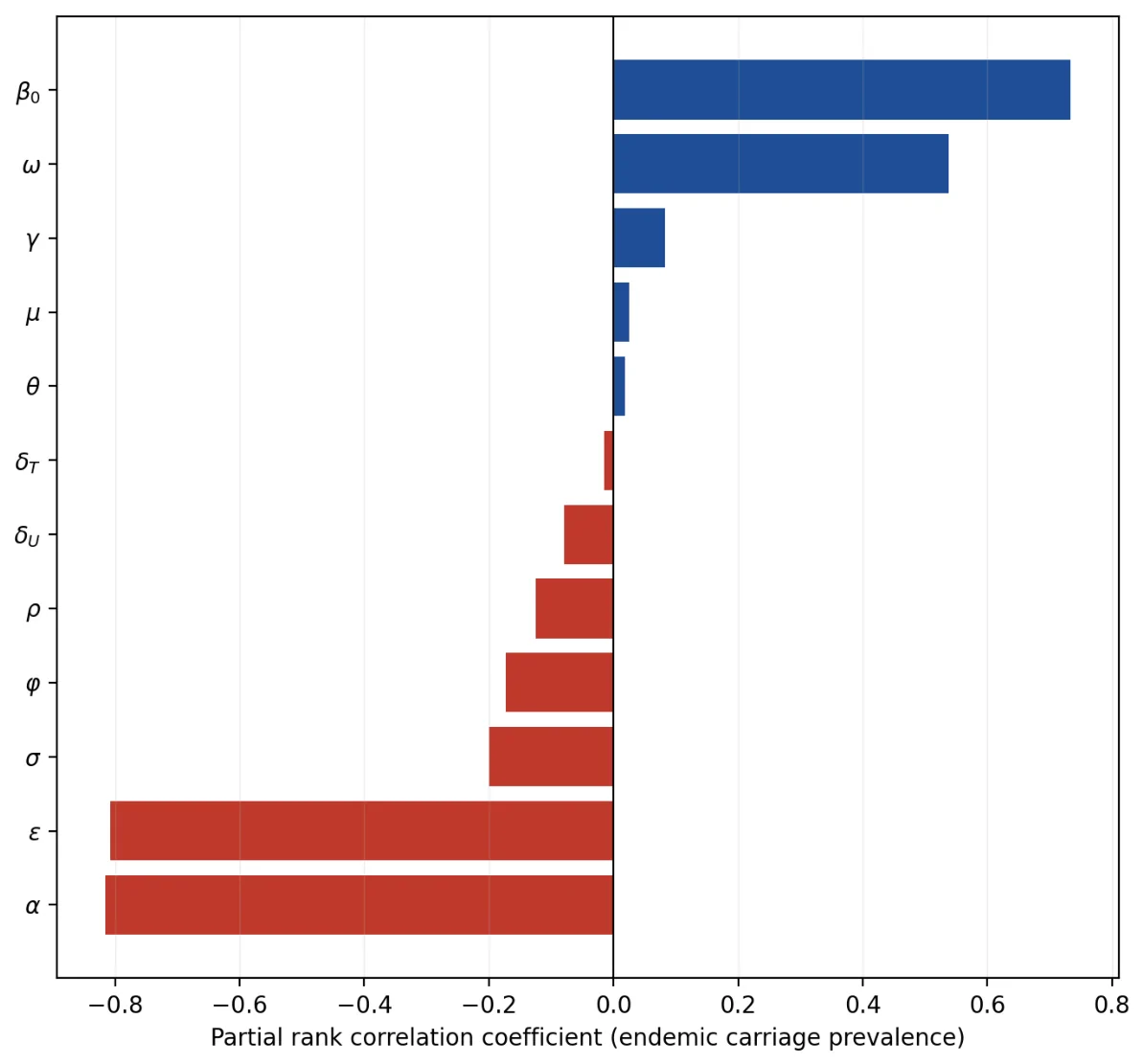
Partial rank correlation coefficients of endemic carriage prevalence with respect to all twelve sampled parameters of Table 1 (Latin-hypercube sample, N=4000, 1710 evaluable points with $R_{0}>1$). Bars are coloured by the sign of the coefficient: blue, positive association (higher parameter value, higher carriage prevalence); red, negative association. Carriage-clearance rate (α), carriage-blocking immunity (ε), mean transmission ($\beta_{0}$), and immunity-waning rate (ω) are jointly the dominant drivers, of comparable magnitude to one another; vaccine efficacy (σ) and routine coverage (φ) are moderate and protective; the remaining parameters (including the case-management parameters $\gamma ,\delta_{T},\delta_{U}$) have small or negligible coefficients (see text for the full ranked set).

The partial-rank-correlation screen in Figure 5—a global screening method that assumes monotonicity between each parameter and the output—ranks the drivers of the (autonomous, unforced) endemic carriage prevalence $C^{*}$ over all twelve parameters of Table 1 simultaneously, computed on a Latin-hypercube sample of N=4000; each equilibrium $C^{*}$ was obtained by direct root-finding on the coupled equilibrium equations (not by long-time integration), and samples with $R_{0}\leq 1$ (no endemic equilibrium; 57% of the sample, mostly at the low-$\beta_{0}$/high-φ end of the sampled range) were excluded, leaving 1710 evaluable points. Every sign accords with the model’s analytical structure. Endemic carriage is governed most strongly by four parameters of comparable magnitude: the carriage-clearance rate α (PRCC ≈−0.82), the carriage-blocking immunity ε (≈−0.81), the transmission rate $\beta_{0}$ (≈+0.73), and natural-immunity waning ω (≈+0.54). The signs of α and $\beta_{0}$ are the numerical signature of Proposition 2 ($R_{0}\approx \beta_{0}/\left(\alpha +\mu \right)$: longer carriage duration and higher contact rate both raise prevalence); ε and ω act by controlling how fast immune individuals return to the transmitting pool ((1−ε)λR re-acquisition and ωR waning respectively), so both raising re-infection risk and faster waning increase prevalence, exactly as Remark 6 describes qualitatively for the bifurcation coefficient—here we find the same two parameters are, quantitatively, also dominant drivers of the prevalence level itself, not only of the local bifurcation direction. Among the remaining, more moderate parameters: vaccine efficacy against carriage σ (≈−0.20) and routine coverage φ (≈−0.17) are both protective, of comparable but smaller magnitude than ε; the invasion rate ρ (≈−0.12) and the untreated fatality hazard $\delta_{U}$ (≈−0.08) have small negative coefficients (through their effect on $K_{C}$ and on the disease-compartment sink, respectively); the recovery rate γ (≈+0.08) has a small positive coefficient, since faster recovery shortens the time invasive cases spend outside the transmitting pool before returning to *R*—consistent with γ and the treated fatality hazard $\delta_{T}$ (≈−0.02) being the two case-management parameters that Corollary 1 shows do enter the local bifurcation condition (via $\alpha^{\prime }$), even though, as here, their quantitative effect on $C^{*}$ itself is small; vaccine waning θ (≈+0.02) and background mortality μ (≈+0.03) are both negligible. $\delta_{U}$’s treated counterpart $\delta_{T}$ and the capacity constant *K* (not separately varied here, since it enters only through δ(I) alongside $\delta_{T},\delta_{U}$) are, consistent with Corollary 1, the parameters with the least effect throughout. Signs and rank order are robust, though magnitudes shift somewhat with sample size and seed.

## 8. Discussion

We have analysed a carriage-structured model of meningococcal meningitis whose minimal structure is nonetheless faithful to two defining features of the disease: transmission is carried by asymptomatic carriage, and invasive disease is, under the transmission structure assumed here, a rare dead end. Three structural conclusions follow.

First, within this model, the reproduction number is a property of carriage and is decoupled from disease incidence: treating notified invasive cases as the transmitting population would estimate the wrong quantity, though this does not mean case-notification data are uninformative about carriage transmission in general (see the Remark following Proposition 2).

Second, the model can exhibit backward bifurcation, and we obtained the local bifurcation direction in closed form: it is governed by a threshold $\varepsilon^{*}$ on carriage-blocking immunity; of the parameters governing recovery and case management after invasion, only the untreated fatality hazard and treatment capacity are provably absent from it, while the invasion rate, recovery rate, and treated fatality hazard all enter it (Corollary 1). We further showed that this bistability is structurally impossible without vaccination but, once any positive routine coverage is present, persists (rather than being confined to some bounded intermediate band) across essentially the entire plausible coverage range (Corollary 2). Numerical continuation confirms a bistable interval of about [0.946,1) at the lower end of the plausible immunity range (ε=0.20)—roughly 5–6% of $R_{0}$ below unity, comparable in width to bistable intervals reported for other partially effective vaccines in the literature we build on [15], though we have not computed the basins of attraction and so cannot say how large a perturbation from the disease-free state is needed to reach the endemic branch (Figure 2). At the baseline value ε=0.55, by contrast, this bistability does not arise at all under routine coverage alone.

Third, under seasonal forcing we searched explicitly, rather than only hypothesising, for the multi-annual recurrence characteristic of the belt, and did not find it: throughout the region of $\left(\beta_{1},\omega \right)$ space we examined, the model settles to a simple annual cycle (Section 6). This is itself an informative negative result: it indicates that a bifurcation-theoretic account of the earlier simulation finding of Irving et al. [9] does not follow from waning immunity and seasonal forcing alone in a model of this structure, and that reproducing their result likely requires an ingredient this model does not include.

These results speak directly to prevention. The parameter that the recent multivalent-vaccine modelling literature identifies as the critical unknown—vaccine effectiveness against carriage [12]—is precisely the quantity σ, and together with the immunity parameter ε it determines whether the disease-free state is the only locally stable outcome or merely one of two coexisting stable states (we have not shown global attractivity of the disease-free state in either regime). Where $\varepsilon <\varepsilon^{*}$—the lower part of the plausible immunity range at routine coverage φ=0.12, though not the baseline value used elsewhere in this paper—driving $R_{0}$ below unity through routine coverage is not sufficient for elimination, and a mass campaign that carries the system into the disease-free basin becomes necessary rather than merely helpful. This gives a dynamical rationale for the SAGE recommendation that routine introduction be paired with catch-up campaigns in high-risk districts [5], and it reframes the “eliminate versus postpone” question as a question about a measurable carriage-immunity threshold.

The model’s central, testable implication is that the quantity a meningitis-belt immunisation programme should track is not case-based incidence or a case-derived $R_{0}$, but a carriage indicator—e.g., periodic nasopharyngeal swab surveys in sentinel age groups, of the kind already used in MenAfriVac impact evaluations [4]—because carriage prevalence, and specifically the vaccine’s measured effect on carriage acquisition (σ) rather than only on case fatality (δ), is what the model identifies as governing both elimination ($R_{0}$) and the risk of stalling just short of elimination (the $\varepsilon^{*}$ threshold). In practice this translates into two concrete recommendations for programme managers and infectious-disease epidemiologists in the belt: (i) post-introduction evaluation of a conjugate vaccine should include a carriage-acquisition endpoint, not case counts alone, since case counts can fall for reasons (case management, health-seeking behaviour, surveillance completeness) unrelated to whether transmission has actually been interrupted; and (ii) where a carriage survey indicates that natural or vaccine-induced protection against re-acquisition remains low even after good routine coverage, our results suggest this is specifically the situation in which a catch-up campaign, rather than further incremental increases in routine coverage, is expected to be needed to reach elimination—a quantitative, if model-based, criterion for that operational decision.

The model is deterministic and homogeneously mixing; it omits age structure, the spatial wave dynamics of belt epidemics, and serogroup competition, each of which bears directly on clinical and public-health applicability rather than being a minor omission. Age structure matters because both carriage prevalence and invasion risk are strongly age-dependent (carriage peaks in school-age children and adolescents, while invasive disease risk is highest in infants and young children [7]), so the age-homogeneous rate φ used in Section 7 cannot, by itself, be mapped onto an age-targeted infant schedule; a genuinely clinically actionable version of this model would stratify S,V,C,I,R by age. Serogroup competition matters because post-MenAfriVac serogroup replacement (the rise of C, W, X, and Y disease noted in the Introduction) means σ, ε, and even $R_{0}$ are properties of a *specific* serogroup or vaccine antigen combination, not of “meningococcal meningitis” as a single entity, so the single-strain model presented here should be read as a template to be replicated per serogroup and coupled through shared carriage-niche competition, rather than as a direct policy tool in its current single-strain form. A further limitation, distinct from the three just discussed, is that Equation (23) treats the seasonal cycle itself as strictly stationary—the same amplitude $\beta_{1}$, mean $\beta_{0}$, and phase ψ repeating identically every year—and so cannot represent secular change in the forcing: a long-term trend or shift in Harmattan-season intensity or duration (whether from inter-annual climate variability or longer-term climate change), a gradual rise in $\beta_{0}$ from urbanisation and increasing population density, or a slow change in health-seeking behaviour or surveillance sensitivity, would all show up in real surveillance data as a change in the pattern superimposed on the seasonal cycle that this model, by construction, has no mechanism to produce. Since Section 6 shows that this model’s seasonal dynamics are already only weakly sensitive to $\beta_{1}$ and ω within a stationary regime, distinguishing genuine multi-annual recurrence from a slow secular drift in the underlying forcing is an empirical question that stationary models of this kind cannot settle, and would need to be addressed with a non-autonomous (time-varying $\beta_{0}\left(t\right)$) extension fitted directly to a multi-decade surveillance series. The invasion rate ρ and the carriage-immunity parameter ε are weakly identifiable from routine surveillance, which is syndromic and only partially serogroup-confirmed; the scenario magnitudes are therefore illustrative rather than calibrated, and should not be read as country-specific forecasts.

The negative finding of Section 6 points to a further, concrete direction: since deterministic seasonal forcing of this carriage-structured model converges to a simple annual cycle throughout the parameter space we searched, reproducing the belt’s irregular multi-annual epidemics evidently requires an ingredient beyond waning immunity and annual forcing. Candidates, in roughly increasing order of structural change, are: demographic and observation stochasticity superimposed on the annual cycle, which can produce apparently irregular multi-annual case patterns even when the underlying deterministic skeleton is period-1; recurrent case importation from neighbouring districts or countries, which can desynchronise an otherwise regular cycle; age structure, which changes the effective mixing and hence the carriage relaxation timescale $1/K_{C}$ relative to the one-year forcing period; and multi-strain serogroup competition, which introduces additional slow variables (serogroup-specific immunity) that could support subharmonic resonance where the single-strain model cannot. We regard stochastic and importation-driven extensions as the most tractable next steps. A final, complementary extension is age structure itself, without which the routine/catch-up scenarios of Section 7 remain abstract rate-and-pulse interventions rather than evaluations of an actual immunisation schedule; a stochastic extension would additionally capture the establishment probability of a spillover and the timing of reactive campaigns relative to the epidemic threshold.

## 9. Conclusions

Meningococcal recurrence in the African meningitis belt is not, on the evidence assembled here, explained by this model as a resonance between annual seasonal forcing and slow immunity dynamics: we searched explicitly for that resonance and did not find it, so we do not offer it as this model’s account of the belt’s multi-annual epidemics (Section 6 and Section 8 discuss what we found instead and what additional structure we think is missing). What the model does establish, sitting atop the (seasonally annual, not multi-annual) carriage system, is that its elimination can be obstructed by bistability when carriage-blocking immunity is weak (specifically at the lower end of the plausible range, not at the baseline value used elsewhere in this paper), and that this bistability is itself a vaccination-induced phenomenon, absent when φ=0 but persisting across essentially the whole plausible routine-coverage range once φ is positive, rather than being confined to a bounded intermediate band (Corollary 2). Because the invasive case is, within this carriage-mediated transmission structure, a dead end for transmission, the reproduction number is a property of carriage and of carriage-clearance/invasion kinetics (α,ρ) alone; the local bifurcation direction, however, is not fully insulated from disease-management parameters, since the recovery rate γ and the treated fatality hazard $\delta_{T}$ enter it through the fraction of invasive cases that return to the immune class (Corollary 1), and only the untreated hazard $\delta_{U}$ and treatment capacity *K* remain provably inert to it. The practical translation is that programme evaluation and research should prioritise carriage-based endpoints over case counts alone. Measuring and improving vaccine efficacy against carriage, and using coverage pulses to carry the reservoir across the threshold, are the levers that our model suggests determine whether conjugate-vaccine programmes end belt epidemics or only defer them—a hypothesis now stated as a testable, carriage-surveillance-based recommendation rather than only as a modelling conclusion.

## Figures and Tables

**Table 1:** Model parameters, baseline values and plausible ranges drawn from the meningitis-belt literature. Rates are per year. Values are illustrative and intended to support structural and scenario analysis, not country-specific calibration.

Symbol	Interpretation	Baseline	Range	Source/Basis
μ	Birth/death rate	0.020	0.017 to 0.025	Belt demography [2,3]
1/α	Mean carriage duration (days)	60	40 to 90	Carriage studies [6,7]
ρ	Invasion rate (per carriage-yr)	0.037	0.02 to 0.06	Case:carrier ratio [6,7]
1/γ	Invasive-disease duration (days)	14	7 to 21	Clinical course [1]
ω	Natural-immunity waning	0.25	0.10 to 1.0	Informed by age-stratified carriage-recurrence patterns [7]; not a direct serological estimate (see Limitations)
θ	Vaccine-protection waning	0.125	0.05 to 0.25	MenAfriVac follow-up [4]
σ	Vaccine efficacy vs. carriage	0.60	0.30 to 0.90	Carriage endpoint [4,12]
ε	Immunity vs. re-acquisition	0.55	0.20 to 0.90	Assumed leaky (see Remark 1); range informed by [7]
φ	Routine vaccination rate	0	0 to 0.30	Programme scenario
$\delta_{T}$	Fatality hazard, treated (yr^−1^)	2.90	1.35 to 4.68	Converted from CFR 10% (5–15%), see below [5]
$\delta_{U}$	Fatality hazard, untreated (yr^−1^)	26.11	17.39 to 60.88	Converted from CFR 50% (40–70%), see below [5]
$\beta_{1}$	Seasonal amplitude	0.5	0.1 to 0.8	Harmattan forcing [3,8]

**Table 2:** Leading complex–eigenvalue pair of the Jacobian at the unforced ($\beta_{1}=0$) endemic equilibrium, baseline parameters, φ=0, ε=0.55, as immunity-waning rate ω varies. Intrinsic period =2π/|Im(λ)|; e-folding time =−1/Re(λ).

$\omega \;({\mathrm{yr}}^{-1})$	Re(λ)	Im(λ)	Period (yr)	E-Folding Time (yr)
1.00	−0.896	1.152	5.5	1.1
0.50	−0.506	0.927	6.8	2.0
0.25	−0.282	0.711	8.8	3.5
0.15	−0.183	0.578	10.9	5.5
0.10	−0.132	0.492	12.8	7.6
0.05	−0.078	0.381	16.5	12.8

**Table 3:** Cumulative burden per $10^{5}$ population over a 25-year horizon under seasonal forcing, from the model of Equation (2). Values are illustrative, computed at the baseline parameters of Table 1.

Scenario	Cumulative Cases	Cumulative Deaths
No vaccination	2302.3	328.6
Routine uptake	496.3	60.8
Routine uptake + coverage pulse	73.5	9.2
Routine uptake + high efficacy	255.7	33.2

## Data Availability

The simulation and numerical-continuation code that generated all figures and the burden table, together with a symbolic verification notebook for the centre-manifold algebra of Section 5, will be deposited in a public repository upon acceptance; in the interim it is available from the author upon request.
